# Kinetic magnetism in triangular moiré materials

**DOI:** 10.1038/s41586-023-06633-0

**Published:** 2023-11-15

**Authors:** L. Ciorciaro, T. Smoleński, I. Morera, N. Kiper, S. Hiestand, M. Kroner, Y. Zhang, K. Watanabe, T. Taniguchi, E. Demler, A. İmamoğlu

**Affiliations:** 1 Institute for Quantum Electronics, ETH Zürich, Zürich, Switzerland; 2 Departament de Física Quàntica i Astrofísica, Facultat de Física, Universitat de Barcelona, Barcelona, Spain; 3 Institut de Ciències del Cosmos, Universitat de Barcelona, Barcelona, Spain; 4 Department of Physics and Astronomy, University of Tennessee, Knoxville, TN USA; 5 Min H. Kao Department of Electrical Engineering and Computer Science, University of Tennessee, Knoxville, TN USA; 6 Research Center for Electronic and Optical Materials, National Institute for Materials Science, Tsukuba, Japan; 7 Research Center for Materials Nanoarchitectonics, National Institute for Materials Science, Tsukuba, Japan; 8 Institute for Theoretical Physics, ETH Zürich, Zürich, Switzerland

**Keywords:** Magnetic properties and materials, Two-dimensional materials

## Abstract

Magnetic properties of materials ranging from conventional ferromagnetic metals to strongly correlated materials such as cuprates originate from Coulomb exchange interactions. The existence of alternate mechanisms for magnetism that could naturally facilitate electrical control has been discussed theoretically^1–7^, but an experimental demonstration^8^ in an extended system has been missing. Here we investigate MoSe_2_/WS_2_ van der Waals heterostructures in the vicinity of Mott insulator states of electrons forming a frustrated triangular lattice and observe direct evidence of magnetic correlations originating from a kinetic mechanism. By directly measuring electronic magnetization through the strength of the polarization-selective attractive polaron resonance^9,10^, we find that when the Mott state is electron-doped, the system exhibits ferromagnetic correlations in agreement with the Nagaoka mechanism.

## Abstract (web summary)

Minimization of kinetic energy leads to ferromagnetic correlations between itinerant electrons in MoSe_2_/WS_2_ moiré lattices even in the absence of exchange interactions.

## Main

Moiré heterostructures of two-dimensional materials provide a platform for the investigation of the physics of strongly correlated electrons. In contrast to well-studied quantum materials, these moiré materials provide a very high degree of tunability of the parameters relevant for controlling correlations, such as carrier density and the ratio of interaction energy to hopping strength. Moreover, unlike cold-atom quantum simulators, the physics and functionality of moiré materials can be varied using readily accessible external electric and magnetic fields, creating a platform in which different many-body phases compete. Since the first realization of a moiré material, a wealth of correlation physics ranging from correlated Mott–Wigner states to the quantum anomalous Hall effect to superconductivity has been observed both in magic-angle-twisted bilayer graphene and in bilayers of transition metal dichalcogenides (TMDs)^11–21^. Except for orbital magnetism in twisted bilayer graphene^15,22^ as well as early spin susceptibility and scanning probe measurements in TMD bilayers^17,23–26^, quantum magnetism in moiré materials has until recently remained experimentally unexplored. Theoretical works have investigated the magnetic properties of the correlated Mott state in a moiré lattice with one electron per lattice site^27,28^ and focused on the possibility of realizing quantum spin liquids^29–31^.

Here we investigate the magnetic properties of electrons in MoSe_2_/WS_2_ heterobilayers using low-temperature confocal microscopy. We focus on magnetization as a function of temperature *T* and out-of-plane magnetic field *B*_*z*_ at dopings around one electron per moiré lattice site (*ν* = 1). For *ν* > 1, our experiments show that the itinerant electrons exhibit a positive Curie–Weiss constant *θ*_CW_. The linear increase in spin susceptibility as a function of the density of doubly occupied sites at *T* ≈ 140 mK is indicative of kinetic ferromagnetic correlations linked to the Nagaoka mechanism^1,2^.

**Fig. 1:**
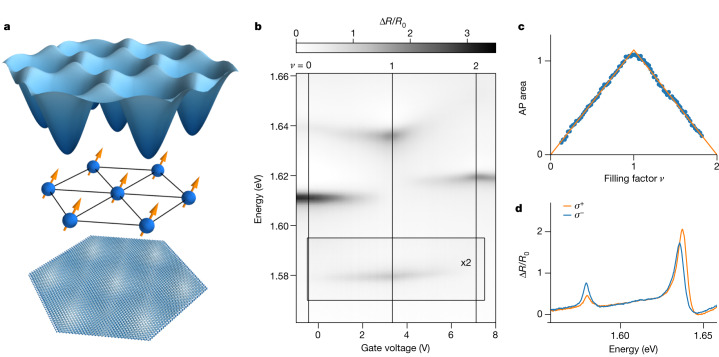
Optical response of the moiré bilayer. **a**, Moiré potential in the conduction band. Electrons occupy the potential minima at the MM sites. **b**, Gate-voltage-dependent normalized reflectance spectrum Δ*R*/*R*_0_ = (*R* − *R*_0_)/*R*_0_, where *R* is the reflection spectrum of MoSe_2_/WS_2_ and *R*_0_ is the background spectrum (see section ‘Background subtraction’). The gate voltage tunes the doping of the heterostructure. At integer fillings, intensity maxima and cusps in the resonance energies appear. **c**, Area of the attractive polaron (AP) resonance as function filling factor at *B*_*z*_ = 0. The linear increase and decrease confirm that electrons occupy a single minimum in the moiré unit cell, forming an isolated Hubbard band. **d**, Polarization-resolved reflection spectrum at *ν* = 1, *B*_*z*_ = 1 T and *T* = 4.2 K. The attractive polaron resonance at 1.58 eV is sensitive to spin polarization of the electrons through its degree of circular polarization. The data in this figure were measured on sample II. Source Data

We study two R-type MoSe_2_/WS_2_ heterostructures encapsulated in h-BN. The lattice mismatch and twist angle between the TMD monolayers create a moiré superlattice with a lattice constant of about 7.5 nm. The minima of the resulting electronic potential for the conduction band are located at high-symmetry points at which the metal atoms in the two layers are aligned (MM sites). The electrons that are injected occupy the triangular lattice of MM sites as shown in Fig. 1a. In sample I, the charge density and the electric field in the heterostructure can be tuned independently using top and bottom graphene gates, whereas sample II is only single-gated.

## Doping-dependent spectrum of MoSe_2_/WS_2_

The reflection spectrum as a function of electron density in Fig. 1b shows several resonances close to the energy of the optical transitions in monolayer MoSe_2_, consistent with previous reports^32,33^. Intensity maxima and cusps in the resonance energies appear at equally spaced gate voltages (see Extended Data Fig. 2 for extended range). These voltages correspond to commensurate filling of the moiré superlattice with one or two electrons per site (*ν* = 1 and 2, respectively), at which incompressible states are formed. We focus here on the resonance at 1.58 eV, which can be identified as an attractive polaron resonance associated with collective excitation of bound electron–exciton pairs (trions) located at the moiré potential minima^9,10,34^. As shown in Fig. 1c, the area, or equivalently the oscillator strength, of the attractive polaron resonance increases linearly as a function of electron density up to filling factor *ν* = 1 and subsequently decreases again linearly between *ν* = 1 and 2.

This behaviour is consistent with the presence of an isolated Hubbard band in which all electrons occupy the same lattice sites, forming doubly occupied sites (doublons) for *ν* > 1 and empty sites (holons) for *ν* < 1. As the attractive polaron resonance is associated with the bound trion of an exciton and a resident electron, it can be optically excited on only moiré lattice sites occupied by a single electron. Consequently, the densities *ν* = *ε* and *ν* = 2 − *ε* provide the same number of sites for attractive polaron formation and hence lead to an identical oscillator strength of the attractive polaron resonance.

The optical selection rules of monolayer MoSe_2_ are retained in the heterostructure, giving rise to circularly polarized resonances for *B*_*z*_ ≠ 0, corresponding to transitions in the K and K′ points of the MoSe_2_ Brillouin zone. The linear dependence of the attractive polaron peak area on the electron density, together with the optical valley selection rules and strong spin–orbit coupling leading to spin–valley locking, enables us to use the polarization-resolved attractive polaron resonance as a quantitative probe of the degree of spin polarization of the electrons. As the attractive polaron is formed by only excitons in the K valley and spin-down electrons in the K′ valley or vice versa^9^, the attractive polaron oscillator strength in *σ*^+^-polarization is proportional to the density *n*_↓_ of spin-down electrons and *σ*^−^-polarization is proportional to the density *n*_↑_ of spin-up electrons. The degree of spin polarization is then given by

1 $${\rho }_{{\rm{s}}}=\frac{{n}_{\uparrow }-{n}_{\downarrow }}{{n}_{\uparrow }+{n}_{\downarrow }}=\frac{{A}_{{\sigma }^{-}}-{A}_{{\sigma }^{+}}}{{A}_{{\sigma }^{-}}+{A}_{{\sigma }^{+}}}=:{\rho }_{{\rm{AP}}},$$

where ${A}_{{\sigma }^{\pm }}$ is the area of the attractive polaron resonance in *σ*^±^ polarization and *ρ*_AP_ denotes the degree of circular polarization of the attractive polaron resonance. The polarization-resolved spectrum in Fig. 1d, measured at *B*_*z*_ = 1 T, *T* = 4.2 K and *ν* = 1, highlights how the attractive polaron resonance becomes partially polarized in a moderate magnetic field. Note that the resonance at 1.635 eV is also sensitive to the spin polarization, mainly through a splitting with giant effective *g*-factor *g*_eff_ = 31, as previously reported for other moiré heterostructures^17,24^.

## Temperature-dependent spin susceptibility

**Fig. 2:**
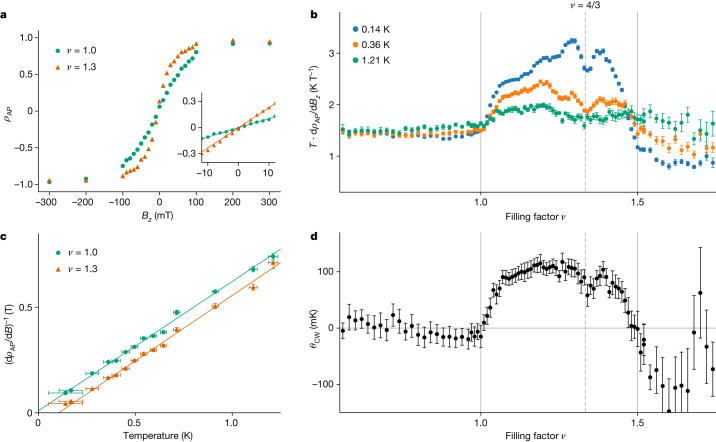
Signatures of kinetic magnetism in single-frequency measurements on sample I at low temperatures. **a**, Degree of polarization *ρ*_AP_ of the attractive polaron resonance as a function of magnetic field *B*_*z*_. Inset, linear fit around *B*_*z*_ = 0 yielding the susceptibility. **b**, Doping dependence of the slope d*ρ*_AP_/d*B*_*z*_ around *B*_*z*_ = 0 and at different temperatures *T*, each multiplied by *T*. A sharp increase in spin susceptibility is observed beyond *ν* = 1, and the susceptibility diverges faster than 1/*T* for 1 < *ν* < 1.5. **c**, Inverse susceptibility as a function of temperature with linear fits yielding the Curie–Weiss constant *θ*_CW_. **d**, Fitted Curie–Weiss constant as a function of *ν*. Vertical error bars correspond to the standard error of the fit (**b**–**d**) and horizontal error bars (**c**) indicate the uncertainty in the temperature measurement (see sections ‘Temperature calibration’ and ‘Curie–Weiss fit’). Source Data

To gain insight into the interactions between spins of the electrons residing in the superlattice potential, we measure the attractive polaron degree of polarization *ρ*_AP_ as a function of *B*_*z*_ for filling factors satisfying 0.5 < *ν* < 1.8. A laser tuned to the peak of the attractive polaron resonance with an excitation power of 11.7 pW is used to avoid light-induced spin depolarization and thereby ensure that we probe magnetic properties of the electronic ground state^35^ (see sections ‘Experimental set-up’ and ‘Power dependence of spin polarization’). We perform a linear fit to extract the slope at *B*_*z*_ = 0, as shown in Fig. 2a, which is related to the magnetic susceptibility through

2 $$\frac{{\rm{d}}}{{\rm{d}}{B}_{z}}{\rho }_{{\rm{AP}}}(\nu )=\frac{{\rm{d}}}{{\rm{d}}{B}_{z}}\frac{M(\nu )}{{M}_{{\rm{s}}}(\nu )}=\frac{\chi (\nu )}{{\mu }_{0}{M}_{{\rm{s}}}(\nu )},$$

where *M*(*ν*) is the magnetization, *μ*_0_ the vacuum permeability and ${M}_{{\rm{s}}}(\nu )=g{\mu }_{{\rm{B}}}{n}_{\nu =1}(1-|\nu -1|)/2$ the saturation magnetization for each density, with *μ*_B_ the Bohr magneton, *g* the MoSe_2_ conduction band *g*-factor and *n*_*ν* = 1_ the electron density at *ν* = 1. The slope d*ρ*_AP_/d*B*_*z*_ around *B*_*z*_ = 0 measured at different temperatures as a function of *ν* is shown in Fig. 2b. Each curve is multiplied by the temperature *T* at which it was measured, such that for paramagnetic behaviour the curves collapse onto one value. The slope is approximately constant for *ν* ≤ 1 and has a sharp linear increase just above *ν* = 1, the point at which the system transitions from a holon-doped to a doublon-doped Mott insulator. A similar sharp decrease occurs at *ν* = 3/2. The enhancement of d*ρ*_AP_/d*B*_*z*_ at low *T* and deviation from 1/*T* behaviour in the range 1 < *ν* < 3/2 are evidence for the presence of ferromagnetic interactions. To quantify the effect, we fit the *T*-dependence of the inverse slope ${\left({\rm{d}}{\rho }_{{\rm{AP}}}/{\rm{d}}{B}_{z}\right)}^{-1}$ with the Curie–Weiss law as shown in Fig. 2c. The resulting doping-dependent Curie–Weiss constant, plotted in Fig. 2d, shows paramagnetic behaviour for *ν* ≤ 1, ferromagnetic interactions for 1 < *ν* < 3/2 and anti-ferromagnetic interactions for *ν* > 3/2. We focus here on the origin of the ferromagnetic correlations.

In general, exchange interactions are expected to play a key part in determining the magnetic order of the system. For a moiré structure with a lattice constant of 7.5 nm, the on-site repulsion is large, leading to small super-exchange interactions *J*_sup_. By contrast, strong Coulomb interactions together with finite overlap of localized Wannier orbitals could ensure that the strength of direct exchange *J*_dir_ exceeds that of *J*_sup_ (ref. ^28^). Using first-principles calculations, we estimate *J*_dir_ = − 0.61 meV (see section ‘Model parameters’). The magnetic properties at *ν* = 1, for which the electrons form an incompressible Mott insulator and are localized on moiré lattice sites, should be exclusively determined by exchange interactions. Surprisingly, we do not find a significant deviation from paramagnetic behaviour at *ν* = 1 (Fig. 2b,d). This suggests that predictions based on density functional theory calculations do not fully capture the physics of our system and exchange interactions do not play a notable part in determining the magnetic properties of electrons. A possible explanation for this is that the electrons are more strongly localized than predicted by theory because of a deeper moiré potential.

Although we cannot rule out a contribution from exchange interactions, the asymmetric behaviour for *ν* = 1 ± *δ* cannot be accounted for by a purely exchange-based mechanism, as it would give rise to similar magnetic interactions for doublon and holon doping. Moreover, a Stoner instability or flat band ferromagnetism would probably lead to signatures peaking at the van Hove singularity located at *ν* = 3/2 for a triangular lattice^36^. By contrast, the ferromagnetic correlations disappear for *ν* ≥ 3/2 in our experiments.

On the basis of these considerations and the fact that strong Coulomb interactions put the moiré structure in the strongly correlated regime of extended Hubbard model physics, we attribute the observed magnetic interactions to the Nagaoka mechanism: in a Hubbard band close to half filling (*ν* = 1), mobile charge carriers can reduce their kinetic energy by inducing magnetic order even in the limit of vanishing exchange interactions^1,2,37^. The linear increase in the Curie–Weiss constant at small doublon doping experimentally agrees with the theoretical prediction of this model^5^. Each injected doublon creates a small ferromagnetic region (magnetic polaron) that results in a linear dependence of ferromagnetic interactions with doping. The dip in the susceptibility and Curie–Weiss constant at *ν* = 4/3 (Fig. 2b,d, dashed line) further corroborates the kinetic nature of the magnetic interactions: at commensurate fractional fillings, the electrons form incompressible Mott–Wigner states^18,25,32^, suppressing the contribution of kinetic energy.

We note that the disappearance of ferromagnetic correlations at *ν* = 3/2 could be associated with the emergence of a spatially ordered structure with a larger period, such as a stripe phase or paired electron crystal. However, treatment of the behaviour at this large doublon density is beyond the scope of our work.

## Theoretical model and numerical analysis

**Fig. 3:**
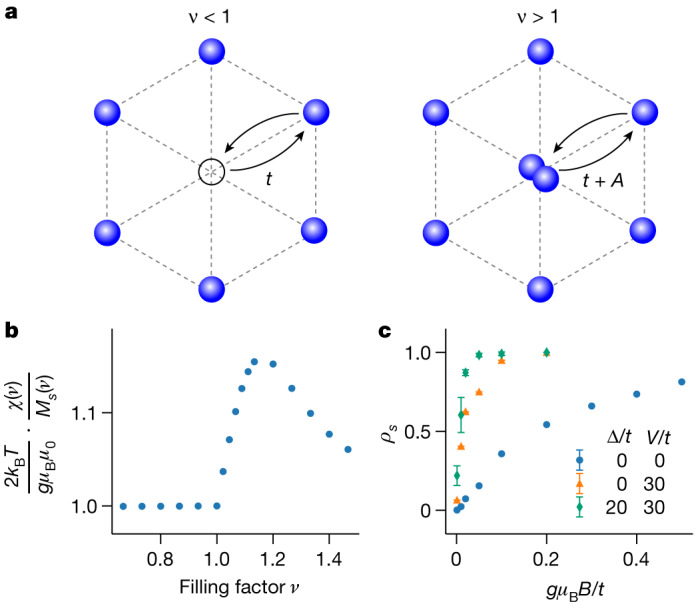
Theoretical analysis of kinetic magnetism strength. **a**, Hopping processes for holons and doublons. The presence of long-range interactions introduces an assisted hopping *A* that modifies the doublon hopping strength. **b**, Simulated spin susceptibility as a function of filling factor for assisted hopping *A* = 10*t* and temperature *k*_B_*T* = *A*, but in the absence of nearest-neighbour Coulomb interaction (*V* = 0), disorder (*Δ* = 0) and exchange interactions (*J*_dir_ = 0 = *J*_sup_). The assisted hopping leads to an asymmetry between doublons and holons. The normalization is chosen such that the value 1 corresponds to a paramagnetic response. **c**, Simulated degree of spin polarization as a function of the magnetic field at *ν* = 0.89 and *T* = 0. Both disorder with distribution width *Δ* and nearest-neighbour Coulomb interactions *V* suppress the anti-ferromagnetic correlations. Error bars correspond to the standard deviation from multiple disorder realizations. Source Data

In a single-band Fermi–Hubbard model on a triangular lattice in the strongly interacting regime, the ferromagnetic interactions for doublon doping induced by the kinetic mechanism are accompanied by anti-ferromagnetic interactions for holon doping^5–7^. To qualitatively understand the experimentally observed absence of anti-ferromagnetic correlations for *ν* < 1, we consider an extended model (see section ‘Theoretical model’) taking into account Coulomb interactions $\widehat{V}$ up to third neighbours, while setting exchange interactions *J*_dir_ and *J*_sup_ to zero, motivated by the paramagnetic response at *ν* = 1. The Coulomb interaction term modifies the hopping of electrons onto sites that are already occupied, which renormalizes the doublon hopping, while leaving the holon hopping *t* unchanged (Fig. 3a). The effective doublon hopping is given by *t* + *A*, with the assisted hopping term $A=-\left.\langle {w}_{i},{w}_{i}\right|\widehat{V}\left|{w}_{i},{w}_{j}\right.\rangle$, where *w*_*i*_ and *w*_*j*_ denote states localized on neighbouring sites^27,28^. Owing to the asymmetry in hopping between holons and doublons, the kinetic magnetism is enhanced for *ν* > 1. Therefore, in an intermediate temperature range *t* ≪ *k*_B_*T* ≲ *t* + *A*, where *k*_B_ is the Boltzmann constant, we expect a sizeable modification of the susceptibility for *ν* > 1, but only negligible deviations from a paramagnetic response for *ν* ≤ 1. This asymmetry of the susceptibility around *ν* = 1 is captured by our finite-temperature tensor network simulations (Fig. 3b). Details on the theoretical model, parameter estimates and the simulations can be found in the Methods.

Furthermore, our calculations show that the overall energy gain from delocalization of holons or doublons is suppressed by the presence of long-range interactions and/or disorder, reducing the strength of kinetic magnetism, particularly for *ν* < 1. In Fig. 3c, we show simulated magnetization curves at *ν* = 0.89 and *T* = 0, comparing the cases with and without long-range interactions or disorder. Introducing disorder or interactions leads to an increased slope at low fields, corresponding to an enhanced susceptibility or suppressed anti-ferromagnetic correlations. This limits the temperatures required to observe kinetic magnetism in the moiré structure to smaller values than would be expected from hopping strengths of the order 1 meV predicted by density functional theory for our moiré structure (see section ‘First-principles simulation with DFT’).

## Conclusion

We demonstrated that the spin susceptibility of electrons in moiré materials can be accurately determined through polarization-resolved attractive polaron oscillator strength measurements at pW power levels. We have used this method to study the filling-factor-dependent spin susceptibility and found a sudden appearance of ferromagnetic correlations for *ν* > 1. Our experimental findings, supported by tensor network simulations, can be attributed to kinetic magnetism in an extended Fermi–Hubbard model on a triangular lattice. Although previous studies found good agreement with ab initio calculations, our observation of a paramagnetic response at *ν* = 1 suggests that direct exchange and super-exchange interactions are weak and the spin physics is dominated by effective kinetic interactions. The strong asymmetry between *ν* < 1 and *ν* > 1 indicates the presence of a large Coulomb-assisted hopping of doublons. Moreover, long-range Coulomb interactions and disorder renormalize the effective hopping for holons and doublons, which in turn ensures that magnetic order in deep moiré potentials exhibiting topologically trivial bands may be observed for only very low temperatures below 100 mK.

During the preparation of this paper, we became aware of several parallel works exploring different aspects of magnetism in bilayer MoTe_2_ moiré structures^38–40^.

## Methods

### Sample fabrication

Graphene, h-BN, MoSe_2_ and WS_2_ layers were exfoliated from bulk crystals onto Si/SiO_2_ (285 nm) substrates and assembled in heterostructures using a standard dry-transfer technique with a poly(bisphenol A carbonate) film on a polydimethylsiloxane (PDMS) stamp. Both samples were encapsulated between two h-BN flakes of 30–40 nm thickness. Optical lithography and electron beam metal deposition were used to fabricate electrodes for the electrical contacts.

### Experimental set-up

**Extended Data Fig. 1:**
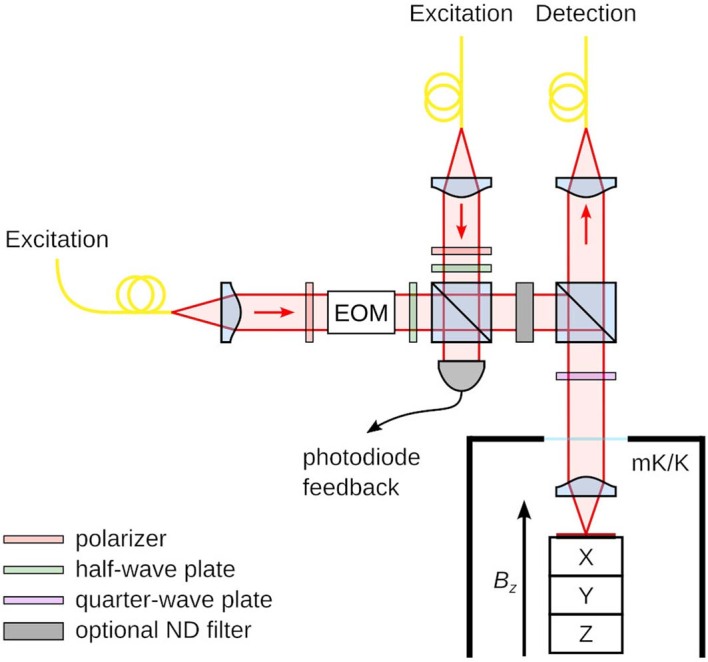
Experimental setup. Schematic of the optical setup used in our experiments.

Experiments on sample I were done in a dilution refrigerator (base temperature ≈ 20 mK) with free-space optical access, with windows at the still, 4 K and room-temperature stages. Sample II was in turn investigated in a liquid helium bath cryostat (also featuring free-space optical access) and in a second dilution refrigerator unit with a single-mode fibre-based optical access (see ref. ^41^ for a detailed description of this set-up). For free-beam set-ups, the samples were mounted on three piezoelectric nanopositioners. A fibre-coupled confocal microscope was used for optical measurements, with either a single aspheric lens or an objective (numerical aperture = 0.7 for both) focusing the light onto a diffraction-limited spot. A schematic of the optical set-up is shown in Extended Data Fig. 1.

Reflection spectra were measured using a supercontinuum laser with a variable filter as a light source and a spectrometer with a liquid-nitrogen- or Peltier-cooled CCD camera as the detector. For mK-temperature measurements of the magnetic circular dichroism (MCD), we used either a tunable continuous wave single-frequency titanium sapphire laser or a few-nm-wide white light with a central wavelength around the attractive polaron resonance. The reflected light was measured using Geiger-mode avalanche photodiodes (APDs) that enable the detection of low-power signals. To reduce the sensitivity of the MCD measurement to slow drifts in the experiments on sample I, the incident light-polarization was switched between *σ*^+^ and *σ*^−^ at kilohertz rates using an electro-optic modulator. By contrast, sample II was illuminated with linearly polarized light. On reflection from the device, the *σ*^+^- and *σ*^−^-polarized components were separated with a polarizing beamsplitter and detected in parallel using two separate APDs. All measurements were power-stabilized with feedback from a photodiode to an acousto-optic modulator or a fibre-coupled variable optical attenuator.

### Background subtraction

Differential reflectance presented in the plots is defined as Δ*R*/*R*_0_ = (*R* − *R*_0_)/*R*_0_, where *R* is the measured reflection spectrum of the heterostructure and *R*_0_ is the background reflection spectrum on the h-BN flakes away from the TMD flakes. For the MCD measurements, the background reflectance ${R}_{0}^{{\sigma }^{\pm }}$ at the laser frequency is measured in both polarizations at charge neutrality or high electron density (*ν* > 2) for which there is no attractive polaron resonance. The degree of circular polarization is then given by

3 $${\rho }_{{\rm{AP}}}=\frac{({R}^{{\sigma }^{+}}-{R}_{0}^{{\sigma }^{+}})-({R}^{{\sigma }^{-}}-{R}_{0}^{{\sigma }^{-}})}{({R}^{{\sigma }^{+}}-{R}_{0}^{{\sigma }^{+}})+({R}^{{\sigma }^{-}}-{R}_{0}^{{\sigma }^{-}})}.$$

### Filling factor calibration

**Extended Data Fig. 2:**
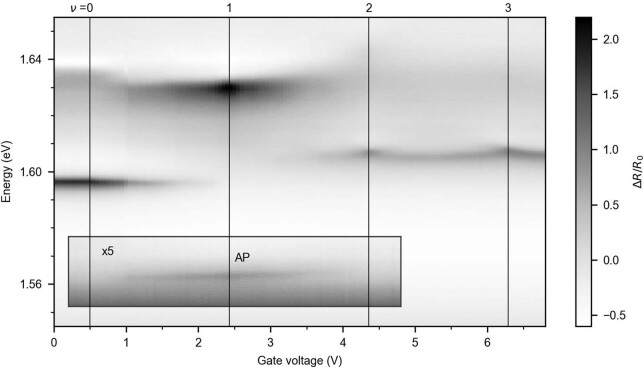
Optical response of the moiré bilayer in an extended voltage range. Normalized reflectance spectrum measured as a function of the applied gate voltage on sample I in an extended voltage range.

We convert the axis of applied gate voltage to the filling factor axis by finding the maxima of the optical resonances for different voltage ranges, which are equally spaced in voltage and peaked at the integer fillings of the moiré lattice (Extended Data Fig. 2). By extracting the positions of the maxima using linear fits (compare Fig. 1c), we estimate the absolute error in the filling factor to be ≤ 0.005.

### Power dependence of spin polarization

**Extended Data Fig. 3:**
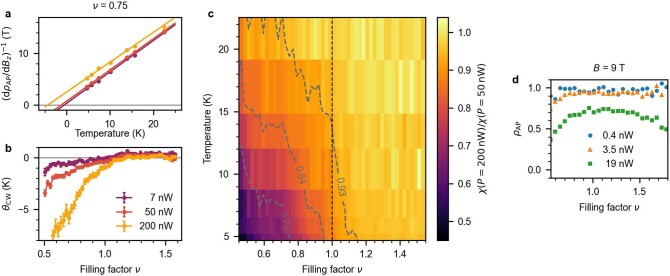
High-temperature measurements of electron spin polarization on sample II. **a**, Inverse slope d*ρ*_AP_/d*B*_*z*_ measured at *ν* = 0.75 as function of temperature for different excitation powers. For each power, the data are precisely reproduced by the Curie–Weiss formula with the Curie–Weiss constant getting sizably lower for larger powers due to light-induced electron spin depolarization. **b**, Fitted CurieWeiss constant as function of electron filling factor for three different excitation powers. For *ν* < 1, the light-induced spin depolarization leads to apparent negative Curie–Weiss constants. Error bars correspond to the standard error of the fit. **c**, Ratio of the slope d*ρ*_AP_/d*B*_*z*_ measured at two different powers, plotted as function of temperature and filling factor. The power dependence changes with both temperature and filling factor and is most prominent at small *ν* and low temperatures. The grey dashed lines mark contours of fixed susceptibility ratios (as indicated). **d**, Degree of polarization measured at fixed magnetic field of *B*_*z*_ = 9 T with different excitation powers and base temperature of the dilution refrigerator. Even at high magnetic field, an excitation power of 19 nW is sufficient at mK temperatures to strongly depolarize the spins and reduce *ρ*_AP_ from 1.0 to 0.7. Source Data

To access the true magnetic ground-state properties of the system, it is essential to ensure that the intensity of the probe light is sufficiently low so as not to perturb the system. Similar to monolayer MoSe_2_ (ref. ^42^), light illumination leads to depolarization of the spin population in the moiré heterostructure. As the strength of the depolarizing effect depends on both temperature and charge density, it can give rise to misleading artefacts in the measured electronic magnetism. This is directly shown by our filling-factor-dependent measurements of the Curie–Weiss constant carried out at high temperatures *T* > 4 K in the bath cryostat on sample II. In these experiments, the sample was illuminated with a white light of tunable power. The magnetic susceptibility of the electron system was extracted based on the degree of circular polarization of the attractive polaron resonance that was, in turn, determined by fitting its spectral profile with a dispersive Lorentzian lineshape^41^. On this basis, we were able to analyse the temperature dependence of the inverse magnetic susceptibility for various filling factors and excitation powers. As seen in Extended Data Fig. 3a (for *ν* = 0.75), although the powers used remain in the sub-μW range, they still markedly affect the magnetic response. More specifically, the Curie–Weiss constant is lower for larger excitation powers. This effect is most prominent for low filling factors and becomes indiscernible at *ν* ≳ 1 (Extended Data Fig. 3b).

This power dependence originates primarily from the changes in spin-valley relaxation dynamics of the electron system. As demonstrated in previous studies of TMD monolayers^43^, the spin relaxation time becomes shorter for larger electron densities and higher temperatures. As a result, if a certain number of electrons undergo a spin flip because of the interaction with optically injected excitons, it takes longer for them to relax back to their ground state when *ν* and *T* are low. For this reason, the magnetic susceptibility determined on exciton injection into the system is lower compared with its unperturbed value. Moreover, the deviation between these two quantities becomes larger for higher excitation powers and lower *ν* and *T* (Extended Data Fig. 3c), which explains the striking power dependence of the Curie–Weiss constant at *ν* < 1 in Extended Data Fig. 3b. In particular, the data in this figure directly show that for the excitons to constitute a nondestructive probe of the electron spin system at *T* > 4 K and 0.5 ≲ *ν* ≲ 1.5, the excitation power needs to be around a few nW.

**Extended Data Fig. 4:**
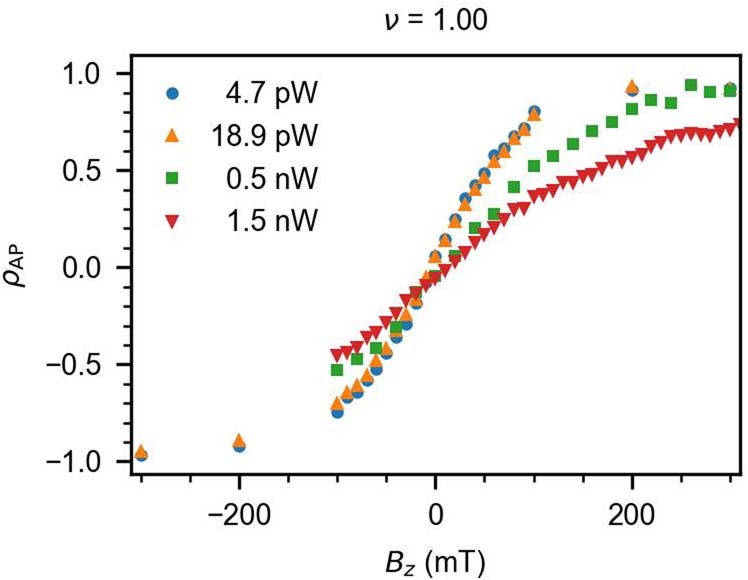
Influence of optical excitation power on low-temperature electron spin polarization. Magnetization curves measured with different excitation powers at *T* ≈ 140 mK in sample I. At nW power levels the magnetization curve is strongly power dependent, indicating that light-induced depolarization is still relevant. The curves measured at pW powers differing by a factor of 4 overlap, demonstrating that the excitation power is sufficiently low to not perturb the system. Source Data

**Extended Data Fig. 5:**
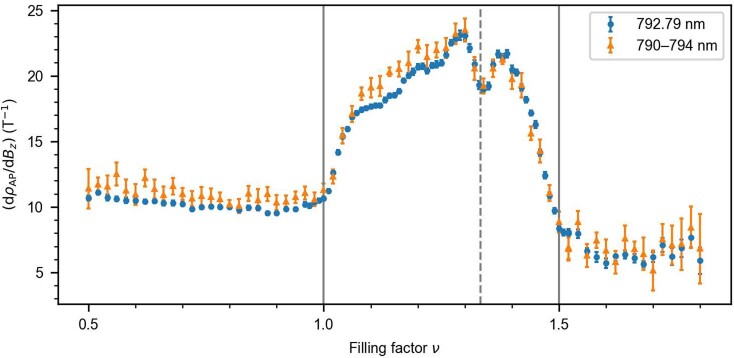
Magnetization measurements using light with different spectral bandwidth. Comparison of the slope d*ρ*_AP_/d*B*_*z*_ measured at base temperature using a single-frequency laser and a broadband source filtered to cover the whole AP resonance. Error bars correspond to the standard error of the fit. Source Data

Owing to the aforementioned temperature dependence of the spin-valley relaxation time, accessing the true magnetic ground-state properties of the electron system at mK temperatures requires us to further reduce the excitation power. As shown in Extended Data Fig. 3d, nW excitation power is sufficient to significantly depolarize the spins at mK temperatures, even in a magnetic field of *B*_*z*_ = 9 T. By measuring magnetization curves at different levels of excitation power (Extended Data Fig. 4), we find that the requisite power for nondestructive probing, for which the power dependence disappears, is of the order of 10 pW. Taking this into account, we used a resonant laser with 11.7 pW incident power on the sample in our mK measurements. Note that this level of power is about six orders of magnitude below the level at which the laser measurably heats the cold finger in the cryostat. Because the line shape and energy of the attractive polaron do not vary appreciably with gate voltage and magnetic field (density-dependent g-factor *g*_AP_ < 10), measuring the reflectance at a single frequency is equivalent to measuring the area of the peak. At constant linewidth, the reflectance at a single frequency is proportional to the area of the whole peak. Line shifts smaller than the linewidth can be tolerated, because they affect only the absolute reflectance, but not the degree of polarization ${\rho }_{{\rm{AP}}}=({R}^{{\sigma }^{+}}-{R}^{{\sigma }^{-}})/({R}^{{\sigma }^{+}}+{R}^{{\sigma }^{-}})$, which is normalized by the total reflectance. We confirm the frequency independence by measurement with a broadband source filtered spectrally to cover the attractive polaron resonance in the whole doping range. The comparison to the single-frequency measurement is shown in Extended Data Fig. 5.

### Effect of optical spin pumping

**Extended Data Fig. 6:**
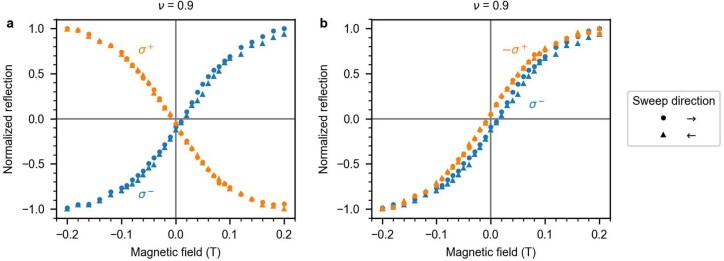
Measurement of the optical spin-pumping effect. **a**, Reflection of a laser (11.7 pW) resonant with the attractive polaron as function of magnetic field measured in both circular polarizations, normalized to lie in the range [ − 1, 1]. The crossing of the two curves occurs at a negative value on the vertical axis, indicating that the magnetization is slightly affected by the laser polarization. **b**, Same data as in **a** but with the *σ*^+^ points mirrored on the horizontal axis. The loop formed by the two curves indicates a small optical spin-pumping effect. Source Data

Excitation with circularly polarized light can lead to an optical spin-pumping effect. Previous studies have shown that this effect is small for MoSe_2_ (ref. ^44^). To exclude that a strong optical spin-pumping effect is present in our system, we measure the magnetic field dependence of the attractive polaron reflectance under the same experimental conditions as in the *ρ*_AP_ measurement, but with fixed circular polarization. We repeat the measurement for both *σ*^+^ and *σ*^−^ polarization. The curves are normalized to the range [−1, 1] and plotted together in Extended Data Fig. 6a. The effect of polarization-dependent optical spin pumping is a vertical displacement of the intersection of the two curves away from 0. We find the intersection is displaced to a negative value, indicating that the polarized laser slightly pumps the spins to the valley it probes, thereby reducing the strength of the probed attractive polaron resonance. In Extended Data Fig. 6b, the same data are plotted with the *σ*^+^ data mirrored on the horizontal axis to better visualize the displacement.

The spin-pumping effect is small, and the measurement of the degree of polarization *ρ*_AP_ is also insensitive to it. As shown in Extended Data Fig. 6, the spin pumping always leads to a slight reduction in the reflectance, regardless of which circular polarization the laser has. Therefore, the effect factors out in the definition of the degree of polarization, ${\rho }_{{\rm{AP}}}=({R}^{{\sigma }^{-}}-{R}^{{\sigma }^{+}})/({R}^{{\sigma }^{-}}+{R}^{{\sigma }^{+}})$.

### Detailed magnetization curves

**Extended Data Fig. 7:**
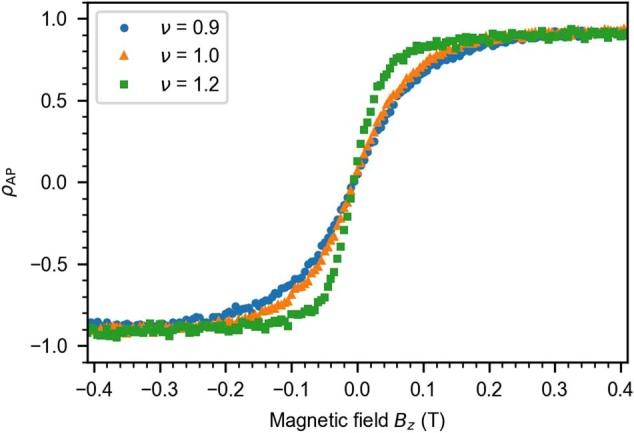
Detailed low-temperature magnetization curves for different electron densities. Degree of polarization *ρ*_AP_ measured as a function of the magnetic field for several filling factors of the moiré lattice. Source Data

Additional plots of *ρ*_AP_(*B*_*z*_) measured at fixed doping in a wider magnetic field range and with smaller step size are shown in Extended Data Fig. 7. The magnetization evolves smoothly with the applied external magnetic field and reaches its saturation value without any discontinuities. No further increase in *ρ*_AP_ is expected at higher magnetic fields, as the curves for all filling factors overlap with that at *ν* = 1.2, for which the ferromagnetic interactions ensure full spin polarization at low fields. The deviation of the saturation value from ±1 arises from difficulties in proper background subtraction for this particular measurement, for which the magnetic field was varied at a fixed filling factor. In the measurements presented in the main text, in which the filling factor was varied at a fixed magnetic field, this problem does not occur and *ρ*_AP_ reaches ±1 at saturation.

### Temperature calibration

Owing to the heat load on our sample through the electrical wiring and finite thermal conductivity at mK temperatures, the real electron temperature of the sample is expected to be higher than the value obtained from the built-in temperature read-out of the dilution refrigerator based on a resistance measurement, especially close to the base temperature. As the electron temperature of the sample is a crucial quantity for our Curie–Weiss analysis, we use the following model to calibrate it and estimate the associated systematic error: The heat transport responsible for cooling the sample is governed by the steady-state heat equation

4 $${\boldsymbol{\nabla }}\left(\kappa {\boldsymbol{\nabla }}T\right)={Q}_{{\rm{in}}},$$

where *κ* is the thermal conductivity and *T* is the temperature, and we assume a constant heat load *Q*_in_ on the sample. At mK temperatures, the heat is transported by electrons through the electrical contacts and wire bonds, so we consider the gradient along only one dimension (along the wire). The electrical conductivity is limited by impurity scattering and therefore independent of temperature, which results in a thermal conductivity proportional to the temperature according to the Wiedemann–Franz law. Setting *κ*(*T*) = *αT*, the equation becomes

5 $$\alpha {\left({T}^{{\prime} }(x)\right)}^{2}+\alpha T(x){T}^{{\prime\prime} }(x)={Q}_{{\rm{in}}}.$$

By integrating twice, we arrive at the solution

6 $$T(x)=\sqrt{\frac{{Q}_{{\rm{in}}}}{\alpha }{x}^{2}+2T(0){T}^{{\prime} }(0)x+T{(0)}^{2}}.$$

**Extended Data Fig. 8:**
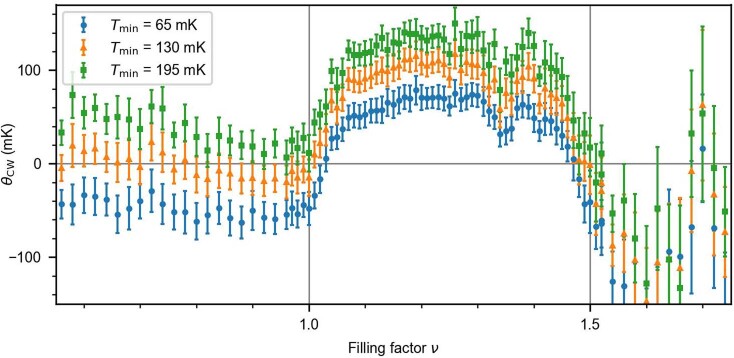
Curie–Weiss temperature determined using a different temperature calibration. Curie–Weiss constant as function of filling factor for different ${T}_{\min }$ used in the temperature calibration. Changing the minimum temperature shifts the curve vertically without affecting it qualitatively. Error bars correspond to the standard error of the fit. Source Data

Using the boundary conditions *T*(0) = *T*_cryo_ (cold-finger temperature according to thermometer read-out) and ${T}^{{\prime} }(0)=0$ (cold finger is well-thermalized), we find the relation

7 $${T}_{{\rm{sample}}}=\sqrt{{T}_{\min }^{2}+{T}_{{\rm{cryo}}}^{2}},$$

where *T*_min_ corresponds to the minimum achievable sample temperature and depends on the heat load and thermal conductivity. A previous independent measurement using a quantum dot in the same cryostat ^45^ found that the sample temperature saturated at *T* = 130 mK. Although the sample and electrical contacts are different, it is reasonable to assume a similar minimum temperature that can be reached in the current measurements. We therefore set *T*_min_ = 130 mK and use equation (7) to convert the temperature read-out to the sample temperature for the Curie–Weiss fit. In Extended Data Fig. 8, we plot the result of the Curie–Weiss fit when different values of *T*_min_ in a plausible range are used, showing the effect of a systematic error in the temperature calibration.

Given that at *θ*_CW_ = 0 at *ν* = 1 within our measurement accuracy, we assume a paramagnetic behaviour at *ν* = 1 to calibrate the sample temperature in the measurements on sample II at *T* > 4 K. For a paramagnet with *J* = 1/2, we have d*ρ*_AP_(*T*)/d*B*_*z*_ = *gμ*_B_/(2*k*_B_*T*), where *μ*_B_ is the Bohr magneton, *k*_B_ the Boltzmann constant and *g* the electronic *g*-factor. The assumption of paramagnetic behaviour at *ν* = 1 is further confirmed by measured magnetization curves that follow ${\rho }_{{\rm{AP}}}({B}_{z})=\tanh (g{\mu }_{{\rm{B}}}{B}_{z}/(2{k}_{{\rm{B}}}T))$. The value *g* = 4.5 of the *g*-factor can be fixed from this relation using the measured magnetization slope at a known temperature, for example, 4.2 K in a helium bath cryostat. We then use the same relation to extract the temperature from the measured slope at *ν* = 1 when heating the sample.

**Extended Data Fig. 9:**
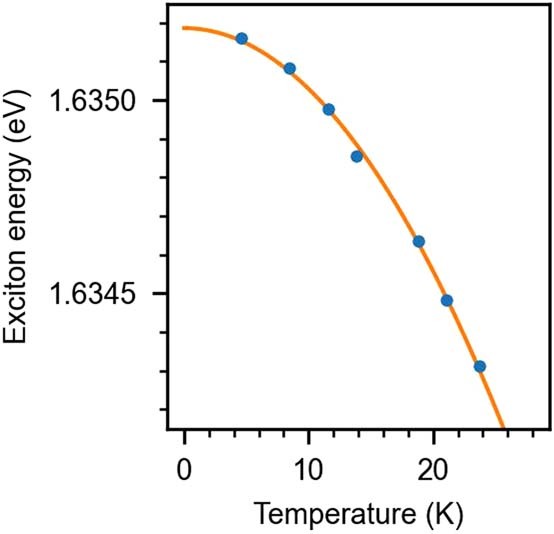
Exciton energy in MoSe_2_ monolayer as a function of temperature. The energy of exciton optical transition measured at charge-neutrality for MoSe_2_ monolayer at different temperatures. The solid line marks the quadratic fit to the data *E*_X_(*T*) = *E*_0_ − *γT*^2^ with *E*_0_ = 1.6352 eV and *γ* = 1.57 *μ*eV/K^2^. Source Data

The temperature values obtained using this method were further verified by analysis of the temperature-induced redshift of the exciton resonance in a MoSe_2_ monolayer region of sample II. As shown in Extended Data Fig. 9, the measured energy *E*_X_(*T*) of this resonance decreases quadratically with temperature, following the Varshni formula *E*_X_(*T*) = *E*_0_ − *γT*^2^ (ref. ^46^). The corresponding *γ* = 1.6 μeV K^−2^ agrees well with the values reported in previous studies of MoSe_2_ monolayers carried out in wider temperature ranges^47^. This finding provides a strong confirmation of the validity of our temperature calibration procedure.

### Curie–Weiss fit

In our measurements, the slope d*ρ*_AP_/d*B*_*z*_ can be measured with very high precision, whereas the sample temperature has a relatively large systematic uncertainty, as described above. To take this into account in the fit, we use the uncertainties in temperature rather than those in susceptibility as weights for the data points. This means that in the linear regression of $a{({\rm{d}}{\rho }_{{\rm{AP}}}/{\rm{d}}{B}_{z})}^{-1}=T-\theta$, the temperature is treated as the dependent variable and the inverse susceptibility as the independent variable — that is, we fit $T=a{({\rm{d}}{\rho }_{{\rm{AP}}}/{\rm{d}}{B}_{z})}^{-1}+\theta$ with 1/*σ*_*T*_ as weights. For the uncertainty *σ*_*T*_ of the temperature, we take ${\sigma }_{T}^{2}={\sigma }_{{\rm{readout}}}^{2}+{({T}_{{\rm{sample}}}-{T}_{{\rm{cryo}}})}^{2}$, where ${\sigma }_{{\rm{readout}}}^{2}$ is the variance of the temperature read-out and the second term quantifies the systematic uncertainty of the temperature as described in the section ‘Temperature calibration’.

### Reproducibility of the low-temperature results on a second device

**Extended Data Fig. 10:**
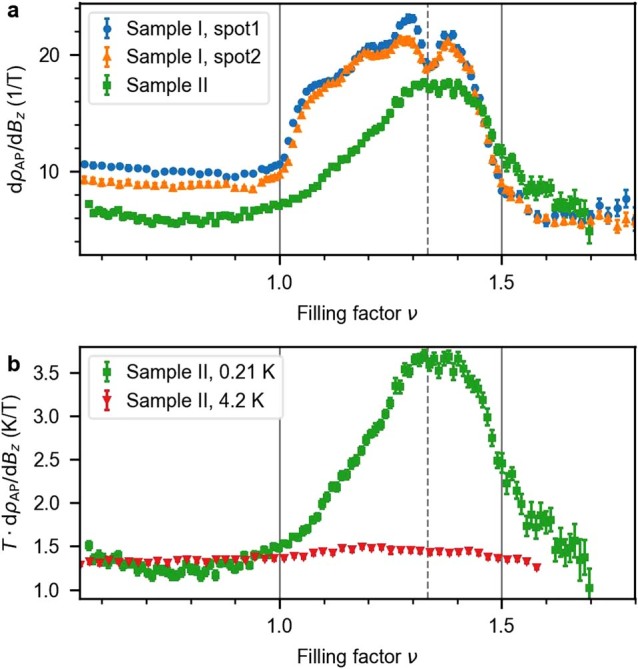
Reproducibility of magnetization data on different spots/samples. **a**, Comparison of the filling factor dependence of d*ρ*_AP_/d*B*_*z*_ measured on two different spots on sample I as well as one spot on sample II at mK temperatures. **b**, Slope d*ρ*_AP_/d*B*_*z*_ measured at base temperature and at 4.2 K on sample II. Similar to Fig. 2b, each curve is multiplied by the temperature *T*, highlighting the enhancement of the magnetic susceptibility at low temperature for 1 < *ν* < 1.5. Error bars correspond to the standard error of the fit. Source Data

The signatures of kinetic magnetism were also observed on two different spots for sample II. For each spot, we measured the degree of circular polarization *ρ*_AP_ of the attractive polaron transition as a function of both electron density and external magnetic field in a second dilution refrigerator unit featuring a base temperature of around 80 mK. On this basis, we determined the filling factor dependence of the slope d*ρ*_AP_/d*B*_*z*_ around *B*_*z*_ = 0. Extended Data Figure 10a shows the results obtained for one of the investigated spots together with data from two spots on sample I. In all three cases, the slope is almost constant at *ν* < 1, starts to increase at *ν* > 1 and finally decreases around *ν* = 1.5. Both of these variations in sample II are markedly less abrupt compared with those seen in sample I. We attribute this difference to a larger disorder of the moiré lattice constant in sample II, caused by an unintentional twist angle of 1.3° in sample II, in contrast to the 0° alignment of sample I. Both twist angles were determined from a calibration of the electron density corresponding to *ν* = 1 based on the Landau-level spacing in monolayer MoSe_2_ regions at high magnetic fields^48^. Owing to the finite twist angle, the moiré lattice constant in sample II is sensitive to variations in the local twist angle, whereas sample I is insensitive to first order. The inhomogeneity of the moiré lattice is responsible for fluctuations of the local filling factor within the examined laser spot. This, in turn, broadens the increase in d*ρ*_AP_/d*B*_*z*_ at *ν* > 1, as the enhancement of magnetic susceptibility due to kinetic magnetism is sensitively dependent on *ν*. The presence of excessive filling factor disorder is independently confirmed by the lack of a robust decrease in d*ρ*_AP_/d*B*_*z*_ around *ν* = 4/3 for sample II, which is due to the formation of a generalized Wigner crystal.

We want to underline that the slightly lower value of d*ρ*_AP_/d*B*_*z*_ at *ν* < 1 in the case of sample II is related to the larger base temperature (≈80 mK) of the second dilution refrigerator unit used for the measurements of this device. This limits the lowest achievable electron temperature, which yields about 210 mK instead of 140 mK for the dilution refrigerator used in the measurements of sample I.

In Extended Data Fig. 10b, we show the same data from sample II together with a measurement at 4.2 K performed in a helium bath cryostat. Similar to Fig. 2b, each curve is multiplied by the temperature of the measurement, highlighting the enhancement of the spin susceptibility for 1 < *ν* < 1.5 at low temperatures with a larger increase than paramagnetic 1/*T* behaviour.

### Theoretical model

To explain the experimental results, we consider a single-band extended *t*–*J* model,

8 $$\begin{array}{cc}\hat{H}= & -t\,\hat{P}\sum _{\langle i,j\rangle ,\sigma }({\hat{c}}_{i,\sigma }^{\dagger }{\hat{c}}_{j,\sigma }+{\rm{h}}.{\rm{c}}.)\hat{P}\\  & +J\sum _{\langle i,j\rangle }\left({{\bf{S}}}_{i}{{\bf{S}}}_{j}-\frac{1}{4}{\hat{n}}_{i}{\hat{n}}_{j}\right)\\  & -\frac{A}{2}\,\hat{P}\sum _{\langle i,j\rangle ,\sigma }[{\hat{c}}_{i,\sigma }^{\dagger }({\hat{n}}_{i,\bar{\sigma }}+{\hat{n}}_{j,\bar{\sigma }}){\hat{c}}_{j,\sigma }+{\rm{h}}.{\rm{c}}.]\hat{P}\\  & +V\sum _{i < j}\frac{{\hat{n}}_{i}{\hat{n}}_{j}}{|i-j|}-h\sum _{i}{\hat{S}}_{i}^{z}+\sum _{i}{\Delta }_{i}{\hat{n}}_{i},\end{array}$$

where ${\hat{c}}_{i,\sigma }$ is the annihilation operator for an electron with spin *σ* on site *i*, ${\hat{n}}_{i}={\sum }_{\sigma }{\hat{n}}_{i,\sigma }={\sum }_{\sigma }{\hat{c}}_{i,\sigma }^{\dagger }{\hat{c}}_{i,\sigma }$ is the electron number operator on site *i*, and ${\vec{{\rm{S}}}}_{i}$ is the electron spin operator on site *i*. The subscripts $\sigma$ and $\bar{\sigma }$ denote opposite electron spins within a sum. The parameter *t* is the hopping strength, *J* is the spin–spin interaction, *A* is the assisted hopping, *V* is the strength of Coulomb interaction projected into the lowest Wannier orbital, *h* is the external magnetic field in units of *gμ*_B_, *Δ*_*i*_ is the on-site potential energy and $\widehat{P}$ is a projector that projects out doublons in the holon-doped regime and holons in the doublon-doped regime. We consider a null spin–spin interaction *J* = 0 motivated by the experimental results pointing to a paramagnetic response at *ν* = 1. Moreover, to implement the long-range coupling proportional to *V*, we cut the range of the interaction at third neighbours. The on-site potential energy *Δ*_*i*_ takes into account spatial variations of the moiré potential. We consider a uniformly distributed disorder *Δ*_*i*_ ∈ [−*Δ*/2, *Δ*/2) of width *Δ* with a corresponding root-mean-square parameter $\Delta /\sqrt{12}$.

### Model parameters

To estimate the relevant parameters of the Hamiltonian used in the tensor network simulations, we start from the finite discrete Fourier expansion of the moiré potential,

9 $$V({\bf{r}})=\mathop{\sum }\limits_{n=1}^{6}{V}_{n}{{\rm{e}}}^{{\rm{i}}{{\bf{G}}}_{n}\cdot {\bf{r}}},$$

where ${V}_{n}=-{V}_{0}\exp \left[{\rm{i}}{(-1)}^{n-1}\varphi \right]$, and we introduce the reciprocal lattice vectors

10 $${{\bf{G}}}_{n}=\frac{4\pi }{{a}_{{\rm{m}}}\sqrt{3}}(\begin{array}{c}\cos (\pi n/3)\\ \sin (\pi n/3)\end{array}),$$

where *a*_m_ is the moiré lattice constant. The parameters *V*_0_ = 6.3 meV and *φ* = 0 are obtained from first-principles calculations.

The single-electron problem is described by the low-energy Hamiltonian,

11 $$\widehat{H}=-\frac{{\hbar }^{2}}{2{m}^{* }}{{\boldsymbol{\nabla }}}^{2}+\widehat{V}({\bf{r}}),$$

where we introduce the effective mass m* = 0.7*m*_e_ of the MoSe_2_ conduction band electrons, where *m*_e_ is the bare electron mass. As the moiré potential has a periodic structure, we can use Bloch’s theorem to write the wavefunctions as

12 $${\psi }_{{\bf{k}}}^{(n)}({\bf{r}})={u}_{{\bf{k}}}^{(n)}({\bf{r}}){{\rm{e}}}^{{\rm{i}}{\bf{k}}\cdot {\bf{r}}},$$

where *n* is the band index, **k** is restricted to the first moiré Brillouin zone (BZ) and ${u}_{{\bf{k}}}^{(n)}$ are the Bloch functions. As the Bloch functions have the same periodicity as the moiré potential, ${u}_{{\bf{k}}}^{(n)}({\bf{r}})={u}_{{\bf{k}}}^{(n)}({\bf{r}}+{{\bf{R}}}_{i})$, we can expand them by performing a discrete Fourier transform

13 $${u}_{{\bf{k}}}^{(n)}({\bf{r}})=\sum _{{\bf{G}}\in {\mathcal{G}}}{c}_{{\bf{k}},{\bf{G}}}^{(n)}{{\rm{e}}}^{{\rm{i}}{\bf{G}}\cdot {\bf{r}}},$$

where ${\mathcal{G}}$ is the set of all reciprocal lattice vectors. Therefore, the Hamiltonian can be written on the basis of reciprocal lattice vectors as

14 $${H}_{{\bf{G}},{{\bf{G}}}^{{\prime} }}({\bf{k}})=\frac{{\hbar }^{2}}{2{m}^{* }}{\left({\bf{k}}+{\bf{G}}\right)}^{2}{\delta }_{{\bf{G}},{{\bf{G}}}^{{\prime} }}+\mathop{\sum }\limits_{n=1}^{6}{V}_{n}{\delta }_{{\bf{G}}-{{\bf{G}}}^{{\prime} },{{\bf{G}}}_{n}},$$

with *δ*_*i,j*_ the Kronecker delta, which can be diagonalized for each quasi-momentum **k** by using a large set of reciprocal lattice vectors. The ground-state solution corresponds to the lowest band *n* = 0. The associated Wannier wavefunction *w*_*i*_(**r**) localized at site **R**_*i*_ is obtained by performing the change of basis

15 $${w}_{i}({\bf{r}})=\frac{1}{\sqrt{{\mathcal{N}}}}\sum _{{\bf{k}}\in {\rm{BZ}}}{\psi }_{{\bf{k}}}({\bf{r}}){{\rm{e}}}^{{\rm{i}}{\bf{k}}\cdot {{\bf{R}}}_{i}},$$

where we drop the band index and introduce the normalization factor ${\mathcal{N}}$.

The interaction potential between charges in the TMDs is given by the Rytova–Keldysh potential^49,50^

16 $${V}_{{\rm{RK}}}(r)=\frac{{e}^{2}}{8{\epsilon }_{0}{r}_{0}}\left({H}_{0}\left(\frac{{\epsilon }_{{\rm{r}}}r}{{r}_{0}}\right)-{Y}_{0}\left(\frac{{\epsilon }_{{\rm{r}}}r}{{r}_{0}}\right)\right),$$

where *H*_0_ is the Struve function, *Y*_0_ the Bessel function of the second kind, *r*_0_ = 3.5 nm the screening length for MoSe_2_, *ϵ*_r_ = 4.5 the relative permittivity of h-BN as the surrounding medium^51^ and *ϵ*_0_ is the vacuum permittivity. The matrix elements

17 $$\begin{array}{lll}t & = & -\left\langle {w}_{i}\right|\widehat{H}\left|{w}_{j}\right\rangle =0.75\,{\rm{meV}}\\ U & = & \left\langle {w}_{i},{w}_{i}\right|{V}_{{\rm{RK}}}(| {{\bf{r}}}_{2}-{{\bf{r}}}_{1}| )\left|{w}_{i},{w}_{i}\right\rangle =157\,{\rm{meV}}\\ V & = & \left\langle {w}_{i},{w}_{j}\right|{V}_{{\rm{RK}}}(| {{\bf{r}}}_{2}-{{\bf{r}}}_{1}| )\left|{w}_{i},{w}_{j}\right\rangle =44.6\,{\rm{meV}}\\ J & = & -\left\langle {w}_{i},{w}_{j}\right|{V}_{{\rm{RK}}}(| {{\bf{r}}}_{2}-{{\bf{r}}}_{1}| )\left|{w}_{j},{w}_{i}\right\rangle =-0.61\,{\rm{meV}}\\ A & = & -\left\langle {w}_{i},{w}_{i}\right|{V}_{{\rm{RK}}}(| {{\bf{r}}}_{2}-{{\bf{r}}}_{1}| )\left|{w}_{i},{w}_{j}\right\rangle =6.1\,{\rm{meV}}\end{array}$$

are evaluated numerically, where $\left|{w}_{i},{w}_{j}\right\rangle$ denotes a state in which two electrons occupy the neighbouring Wannier orbitals.

### Tensor network simulations

Our finite-temperature tensor network simulations are based on a purification scheme performed in the canonical ensemble. We implement the *U*(1) symmetry associated with the conservation of the total number of electrons, but we do not fix the net magnetization of the system. The finite-temperature density matrix is represented as a matrix product state (MPS) in a doubled Hilbert space. The MPS maximum bond dimension is set to *χ* = 768. The cooling process is performed as indicated in ref. ^5^. We progressively apply an infinitesimal (*δβ* = 0.1) Boltzmann factor e^−*δβ*/2^ by using the *W*_II_ technique^52^. The finite-temperature calculations are performed in a triangular cylinder of size *L* = *L*_*x*_ × *L*_*y*_ = 15 × 3.

To obtain the ground state of the system, we use the density-matrix renormalization group algorithm. We perform simulations in a triangular cylinder of size *L* = *L*_*x*_ × *L*_*y*_ = 15 × 6, and we fix the maximum bond dimension of our MPS to *χ* = 1,024. To capture the effects of a disordered on-site potential, we have performed calculations in three different disorder realizations and taken the average. The tensor network calculations have been performed using TeNPy (ref. ^53^).

### First-principles simulation with DFT

We study TMD heterobilayers with a small twist angle starting from R-stacking, in which every metal (M) or chalcogen (X) atom on the top layer is aligned with the same type of atom on the bottom layer. In a local region of a twisted bilayer, the atom configuration is identical to that of an untwisted bilayer, in which one layer is laterally shifted relative to the other layer by a corresponding displacement vector ***d***_0_. Therefore, the moiré band structures of twisted TMD heterobilayers can be well described by the continuum model.

In particular, ${{\bf{d}}}_{0}=0,-\left({{\bf{a}}}_{1}+{{\bf{a}}}_{2}\right)/3,\left({{\bf{a}}}_{1}+{{\bf{a}}}_{2}\right)/3$, where **a**_1,2_ is the primitive lattice vector of untwisted bilayers, corresponding to three high-symmetry stacking configurations of untwisted TMD bilayers, which we refer to as MM, XM and MX. In MM stacking, the M atom on the top layer is locally aligned with the M atom on the bottom layer, whereas in MX stacking, the M atom on the top layer is locally aligned with the X atom on the bottom layer, likewise for XM. The bilayer structure in these stacking configurations is invariant under three-fold rotation around the *z*-axis.

The density functional theory (DFT) calculations are performed using the generalized gradient approximation with SCAN density functional^54^ with dDsC dispersion correction method, as implemented in the Vienna Ab initio Simulation Package. We note that SCAN + dDsC captures the intermediate-range van der Waals interaction through its semi-local exchange term. Pseudo-potentials are used to describe the electron–ion interactions. We first construct the rigid structure of an R-stacked MoSe_2_/WS_2_ heterobilayer with vacuum spacing larger than 20 Å to avoid artificial interaction between the periodic images along the *z*-direction. The lattice constants 3.32 Å and 3.19 Å are taken from bulk structures for MoSe_2_ and WS_2_, respectively. The structure relaxation is performed with force on each atom less than 0.01 eV Å^−1^. We use 1 × 1 × 1 for structure relaxation and self-consistent calculation because of the expensive computational cost.

**Extended Data Fig. 11:**
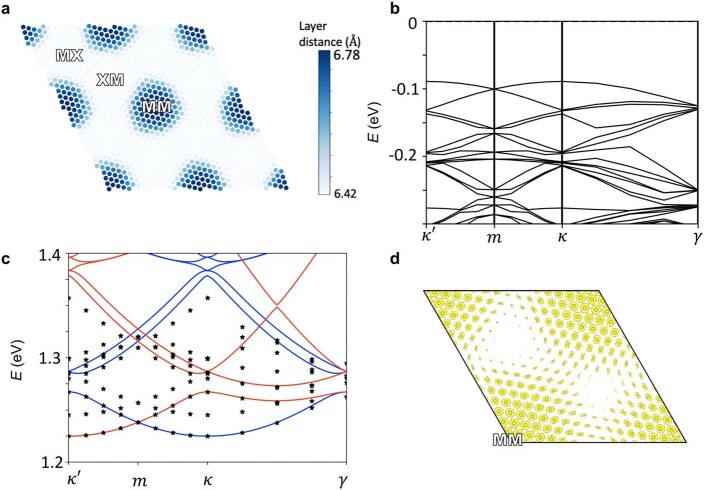
DFT simulations of the electronic band structure. **a**, The interlayer distance of the 2 × 2 R-stacked MoSe_2_/WS_2_ obtained from DFT is shown, demonstrating a large variation between the MM and XM/MX regions. **b**, Projected moiré valence band structures from large scale DFT calculations at twist angle *θ* = 2.7°. **c**, The continuum model band structure (blue and red lines for K and K′ valley) is plotted in comparison with large scale DFT calculations (black dots) at twist angle *θ* = 2.7°, showing excellent agreement. **d**, *γ* momentum wavefunction plot for lowest moiré conduction band, localizing at MM regions in the MoSe_2_ layer.

For R-stacked MoSe_2_/WS_2_, we find that lattice relaxation is significant, which is the main source for the moiré potential. Our DFT calculations at *θ* = 2.7° with 1,545 atoms per unit cell show a significant variation of the layer distance *d* in different regions on the moiré superlattice (Extended Data Fig. 11a). The smallest distance *d* = 6.42 Å is in MX and XM stacking regions, in which a metal atom on the top layer is aligned with a chalcogen atom on the bottom layer and vice versa, whereas the largest distance *d* = 6.78 Å is in MM region, in which metal atoms of both layers are aligned. With the fully relaxed structure, the low-energy moiré conduction bands of R-stacked MoSe_2_/WS_2_ are found to come from the ±K valley of MoSe_2_ after applying a gating field *E* = 0.5 V nm^−1^, to be consistent with experimental observations.

From the fitting of moiré conduction bands, we obtain the continuum model parameters of the lowest bands as *V*_0_ = 6.3 meV, *φ* = 0°, with the bulk lattice constant *a*_0_ = 3.32 Å. Therefore, the effective model for the moiré conduction band is an ideal triangular lattice Hubbard model, with the MM region as the single potential minimum (Extended Data Fig. 11d) for the wavefunction plot. To demonstrate the accuracy of the continuum model method, we compare the conduction band structures computed by large-scale DFT directly at *θ* = 2.7° and by the continuum model (Extended Data Fig. 11c). We note that the DFT-calculated spin–orbit splitting of the conduction bands is 22 meV, whereas the bandwidth of the lowest moiré band extracted from continuum model is 10 meV for a twist angle of 0°. As for the moiré valence band, the continuum model parameters can be fitted as *V*_0_ = 1.9 meV and *φ* = 59^∘^, with nearly identical moiré potential at the MM and MX regions.

## Online content

Any methods, additional references, Nature Portfolio reporting summaries, source data, extended data, supplementary information, acknowledgements, peer review information; details of author contributions and competing interests; and statements of data and code availability are available at 10.1038/s41586-023-06633-0.

### Source data

Source Data Fig. 1

Source Data Fig. 2

Source Data Fig. 3

Source Data Extended Data Fig. 3

Source Data Extended Data Fig. 4

Source Data Extended Data Fig. 5

Source Data Extended Data Fig. 6

Source Data Extended Data Fig. 7

Source Data Extended Data Fig. 8

Source Data Extended Data Fig. 9

Source Data Extended Data Fig. 10

## Data Availability

The data that support these findings are available at the ETH Research Collection (http://hdl.handle.net/20.500.11850/610987). Source data are provided with this paper.

## Code availability

The code used for tensor network simulations and DFT calculations is available from the corresponding author on reasonable request.
